# Stimulation of the Sigma-1 Receptor by DHEA Enhances Synaptic Efficacy and Neurogenesis in the Hippocampal Dentate Gyrus of Olfactory Bulbectomized Mice

**DOI:** 10.1371/journal.pone.0060863

**Published:** 2013-04-08

**Authors:** Shigeki Moriguchi, Yasuharu Shinoda, Yui Yamamoto, Yuzuru Sasaki, Kosuke Miyajima, Hideaki Tagashira, Kohji Fukunaga

**Affiliations:** Department of Pharmacology, Graduate School of Pharmaceutical Sciences, Tohoku University, Sendai, Japan; Chiba University Center for Forensic Mental Health, Japan

## Abstract

Dehydroepiandrosterone (DHEA) is the most abundant neurosteroid synthesized *de novo* in the central nervous system. We previously reported that stimulation of the sigma-1 receptor by DHEA improves cognitive function by activating calcium/calmodulin-dependent protein kinase II (CaMKII), protein kinase C and extracellular signal-regulated kinase in the hippocampus in olfactory bulbectomized (OBX) mice. Here, we asked whether DHEA enhances neurogenesis in the subgranular zone of the hippocampal dentate gyrus (DG) and improves depressive-like behaviors observed in OBX mice. Chronic treatment with DHEA at 30 or 60 mg/kg p.o. for 14 days significantly improved hippocampal LTP impaired in OBX mice concomitant with increased CaMKII autophosphorylation and GluR1 (Ser-831) phosphorylation in the DG. Chronic DHEA treatment also ameliorated depressive-like behaviors in OBX mice, as assessed by tail suspension and forced swim tests, while a single DHEA treatment had no affect. DHEA treatment also significantly increased the number of BrdU-positive neurons in the subgranular zone of the DG of OBX mice, an increase inhibited by treatment with NE-100, a sigma-1 receptor antagonist. DHEA treatment also significantly increased phosphorylation of Akt (Ser-473), Akt (Ser-308) and ERK in the DG. Furthermore, GSK-3β (Ser-9) phosphorylation increased in the DG of OBX mice possibly accounting for increased neurogenesis through Akt activation. Finally, we confirmed that DHEA treatment of OBX mice increases the number of BrdU-positive neurons co-expressing β-catenin, a downstream GSK-3βtarget. Overall, we conclude that sigma-1 receptor stimulation by DHEA ameliorates OBX-induced depressive-like behaviors by increasing neurogenesis in the DG through activation of the Akt/GSK-3β/β-catenin pathway.

## Introduction

Alzheimer’s disease (AD) patients show severe impairment of the olfactory system [1], [2], [3], which occurs at early stages of AD [4]. The olfactory bulb is innervated from the medial septum, which is enriched in choline acetyltransferase-positive neurons. Likewise, AD patients show cholinergic degeneration in the medial septum, which may underlie impaired olfactory function. Olfactory bulbectomy (OBX) in mice causes retrograde degeneration of cholinergic neurons in the medial septum, thereby reducing cholinergic activity in the hippocampus and cortex [5]. In fact, rats subjected to OBX show abnormal behaviors such as cognitive deficits [6], depressive-like behaviors [7], muricide [8], and increased exploratory behaviors [9]. We previously reported that OBX mice show impaired long-term potentiation (LTP) and down-regulation of calcium/calmodulin-dependent protein kinase II (CaMKII) and protein kinase C (PKC) activities in the hippocampal CA1 region [10].

Notably, depressive-like behaviors are associated with impaired neurogenesis in the subgranular zone of the hippocampal dentate gyrus (DG) in rodents [11].

Anti-depressants such as fluoxetine or imipramine improve impaired neurogenesis in the rodent DG [12], [13]. In fact, impaired neurogenesis in the DG has been observed in OBX mice [14], a deficit associated with cognitive decline [15]. For example, suppressed DG neurogenesis by X-irradiation leads to impaired hippocampal-dependent memory in rodents [16], [17]. By contrast, in rodents X-irradiation of a restricted region of hippocampus prevents neurogenesis and antagonizes amelioration of depressive-like behaviors by anti-depressants [13].

Dehydroepiandrosterone (DHEA), one of the most abundant endogenous neuroactive steroids, is synthesized *de novo* from cholesterol and secreted either from the adrenal gland or by cells in the CNS [18]. In humans, DHEA blood levels increase throughout childhood but markedly decrease in the aged [19], [20]. Thus, age-dependent changes in DHEA levels may trigger the onset of age-related cognitive deficits, including AD [21]. Notably, DHEA acts to promote anti-amnesic effects through stimulation of the sigma-1 receptor (sigma-1R) [22]. We also reported that repeated DHEA treatments ameliorate cognitive deficits seen in OBX mice through activation of sigma-1R [23].

Hippocampal LTP is the molecular mechanism underlying cognitive function [24]. We previously reported that LTP induction is regulated by several protein kinases including CaMKII and calcium/calmodulin-dependent protein kinase IV (CaMKIV) [25], [26]. CaMKII is highly expressed in post-synaptic densities of excitatory synapses and becomes constitutively active through autophosphorylation during LTP induction [25], [27], [28], [29]. During enhancement of synaptic efficacy, CaMKII autophosphorylation promotes recruitment of the α-amino-3-hydroxy-5-methyl-4-isoxazolpropionic acid receptor (AMPAR) into the synaptic membrane [30], [31], [32] and potentiates AMPAR function by phosphorylation of GluR1 [28]. By contrast, CaMKIV is primarily expressed in neuronal nuclei [33], [34]. CaMKIV activation is associated with LTP induction through up-regulation of phosphorylation of the transcription factor cyclic AMP-responsible element-binding protein (CREB) [26].

In this present study, we found that sigma-1R stimulation by DHEA in the hippocampal DG ameliorates depressive-like behaviors through by enhancing neurogenesis via activation of the protein kinase B (Akt)/glycogen synthase kinase-3 beta (GSK-3β)/β-catenin pathway.

## Results

### DHEA Ameliorates LTP in the DG of OBX Mice

**Figure 1:**
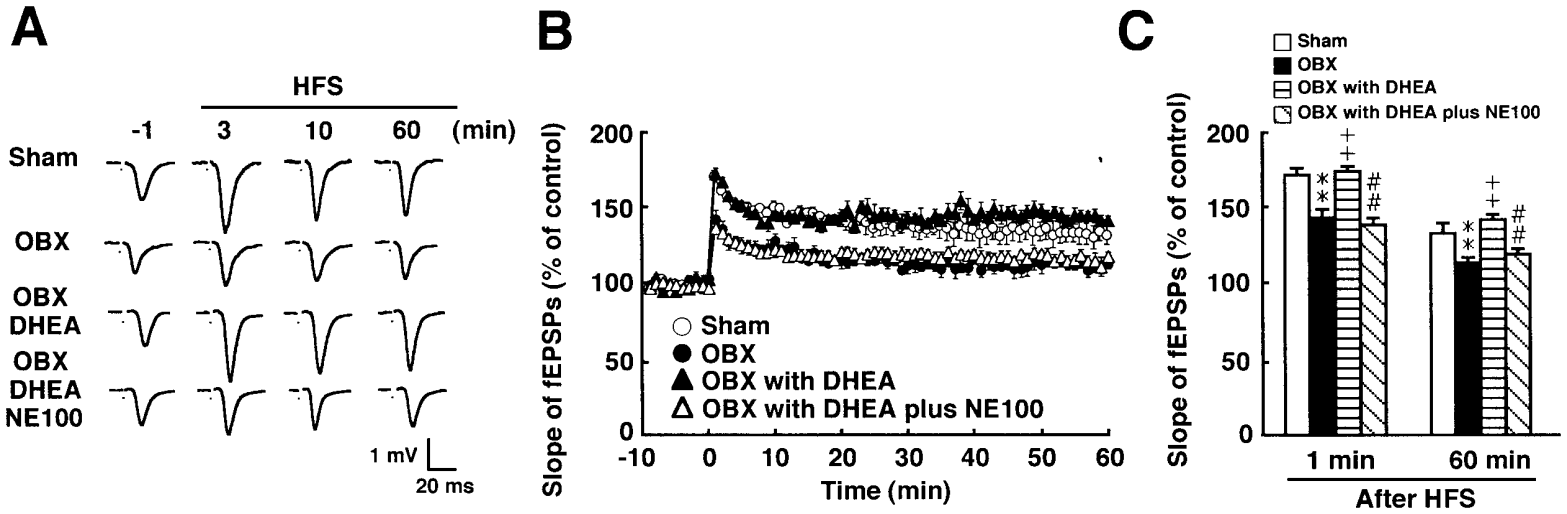
DHEA ameliorates perturbed LTP in the DG of OBX mice. A: Representative fEPSPs were recorded from the DG in sham-operated mice, OBX mice, DHEA (60 mg/kg p.o.)-treated OBX mice, and DHEA plus NE-100 (1 mg/kg p.o.)-treated OBX mice. B: Changes in fEPSP slope following HFS recorded in the DG were attenuated in OBX compared with sham-operated mice. DHEA treatment significantly improved LTP in OBX mice. NE-100 significantly inhibited enhancement of LTP mediated by DHEA in OBX mice. C: Changes in fEPSP slope following HFS are shown in sham-operated, OBX, DHEA-treated OBX, and DHEA plus NE-100-treated OBX mice at 1 or 60 min. Vertical lines show SEM. **, p<0.01 versus sham-operated mice. ^++^, p<0.01 versus OBX mice. ##, p<0.01 versus DHEA-treated OBX mice.

We previously documented that stimulation of sigma-1R by DHEA ameliorates impaired LTP in the hippocampal CA1 region of OBX mice [23]. Thus, we initially asked whether repeated DHEA administration improves impaired LTP in the DG as it does in the CA1 region. To do so, we analyzed LTP in the DG obtained from sham-operated mice and from OBX mice with or without treatment with DHEA or DHEA plus the sigma-1R antagonist NE-100. For these studies, mice were treated for 13–14 days with DHEA or DHEA plus NE-100 starting 14 days after OBX surgery. In control slices from sham-operated mice, high frequency stimulation (100 Hz, 2 trains) of the SGZ induced LTP in the DG, which lasted over 60 min (133.1±6.4% of the baseline at 60 min, n = 5) (Fig. 1A, 1B and 1C). As reported previously, markedly reduced LTP was observed in OBX compared with sham-operated mice (113.4±3.1% of the baseline at 60 min, n = 5) (Fig. 1A, 1B and 1C). DHEA administration (60 mg/kg) significantly improved LTP in the OBX mouse DG (142.5±2.5% of baseline at 60 min, n = 5) (Fig. 1A, 1B and 1C). Furthermore, pre-administration of NE-100 (1 mg/kg) significantly inhibited improvement of LTP by DHEA in the DG (119.5±3.2% of baseline at 60 min, n = 5) (Fig. 1A, 1B and 1C).

### DHEA Treatment Increases CaMKIIα (Thr-286) Autophosphorylation and GluR1 (Ser-831) Phosphorylation in the DG of OBX Mice

**Figure 2:**
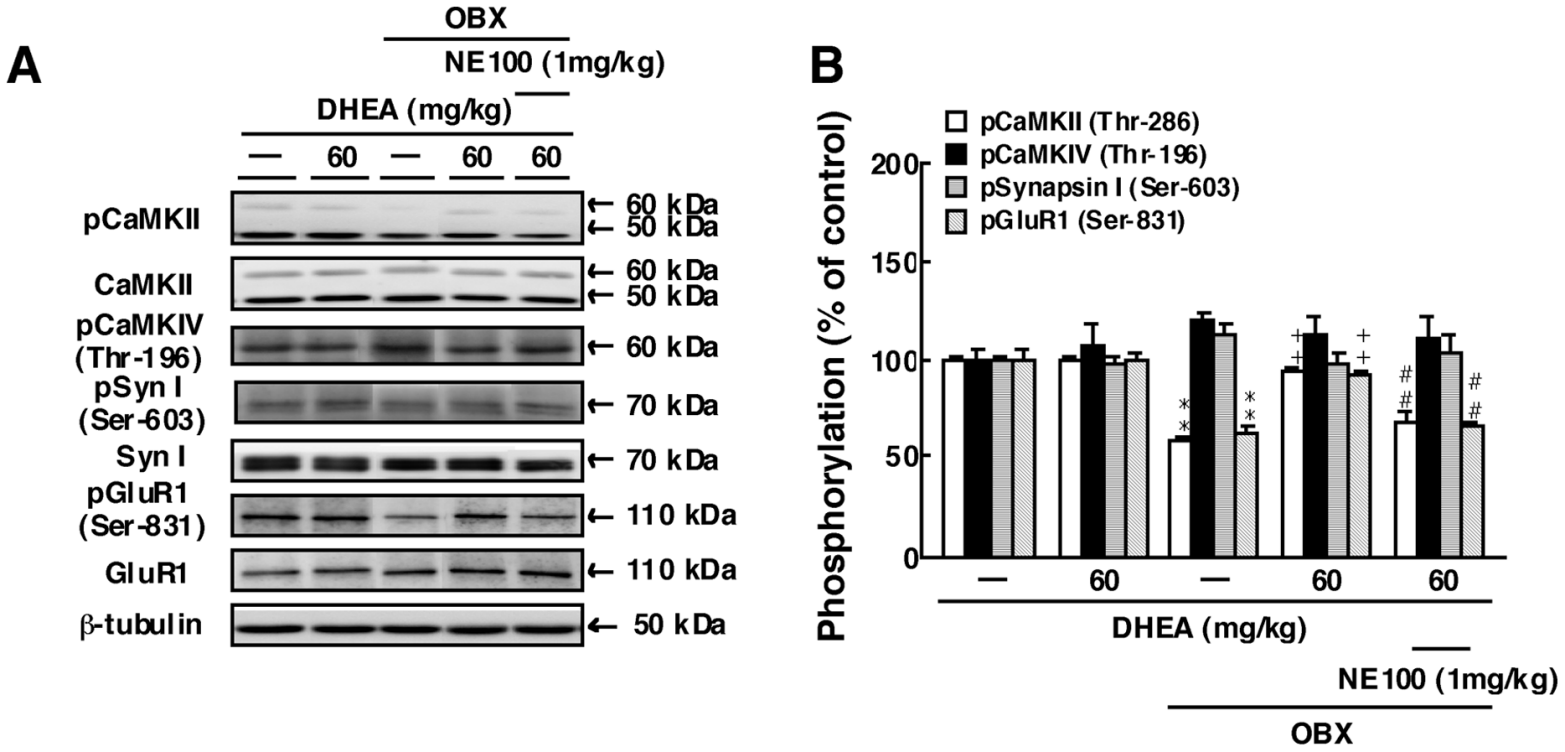
DHEA rescues reduced autophosphorylation of CaMKIIα (Thr-286) and phosphorylation of GluR1 (Ser-831) but does not alter phosphorylation of CaMKIV (Thr-196) or synapsin I (Ser-603) in the DG of OBX mice. A: Representative images of immunoblots using antibodies against autophosphorylated CaMKII, CaMKII, phosphorylated CaMKIV (Thr-196), phosphorylated synapsin I (Ser-606), synapsin I, phosphorylated GluR1 (Ser-831) and GluR1. B: Quantitative analyses of autophosphorylated CaMKIIα (Thr-286) and phosphorylated CaMKIV (Thr-196), synapsin I (Ser-606) and GluR1 (Ser-831). Vertical lines show SEM. **, p<0.01 versus sham-operated mice. ^++^, p<0.01 versus OBX mice. ##, p<0.01 versus DHEA-treated OBX mice.

To define signals activated by DHEA-mediated sigma-1R stimulation in OBX mice, we examined the effect of DHEA administration on autophosphorylation of CaMKIIα (Thr-286) and phosphorylation of CaMKIV (Thr-196) (Fig. 2). In sham-operated mice, repeated DHEA administration (60 mg/kg) had no effect on CaMKIIα autophosphorylation or phosphorylation of CaMKIV (Thr-196), synapsin I (Ser-603) or GluR1 (Ser-831) in the DG. In untreated OBX mice, CaMKIIα (Thr-286) autophosphorylation in the DG markedly decreased compared to sham animals (59.3±2.4% of sham-operated mice, n = 4). DHEA treatment at 60 mg/kg significantly increased CaMKIIα (Thr-286) autophosphorylation in the DG from these OBX mice (94.1±2.0% of sham-operated mice, n = 4) (Fig. 2A and 2B). Pre-treatment with NE-100 significantly inhibited increased CaMKIIα (Thr-286) autophosphorylation mediated by DHEA in the DG of OBX mice (69.3±6.1% of sham-operated mice, n = 4). OBX surgery and/or DHEA treatments did not alter expression levels of either the α or β subunits of CaMKII protein (Fig. 2A). By contrast, CaMKIV phosphorylation (Thr-196) was not altered by either OBX surgery or DHEA treatment (n = 4) (Fig. 2A and 2B).

We next examined phosphorylation of synapsin I (Ser-603), as a downstream target of pre-synaptic CaMKII and GluR1 (Ser-831) as downstream target of post-synaptic CaMKII in the DG [41], [42]. Like CaMKIIα (Thr-286) autophosphorylation, GluR1 (Ser-831) phosphorylation markedly decreased in the DG from OBX mice (62.3±3.8% of sham-operated mice, n = 4) (Fig. 2A and 2B). DHEA treatment at 60 mg/kg significantly increased GluR1 (Ser-831) phosphorylation in the DG of OBX mice (92.9±1.7% of sham-operated mice, n = 4) but not in sham-operated mice (Fig. 2A and 2B). NE-100 pre-treatment significantly inhibited increased GluR1 (Ser-831) phosphorylation by DHEA in the DG of OBX mice (67.1±2.5% of sham-operated mice, n = 4). By contrast, phosphorylation of synapsin I (Ser-603) was not altered by OBX surgery or DHEA treatment (n = 4). OBX surgery and/or DHEA treatments did not alter expression levels of GluR1 and synapsin I proteins (Fig. 2A).

### DHEA Ameliorates Depressive-like Behaviors in OBX Mice

**Figure 3:**
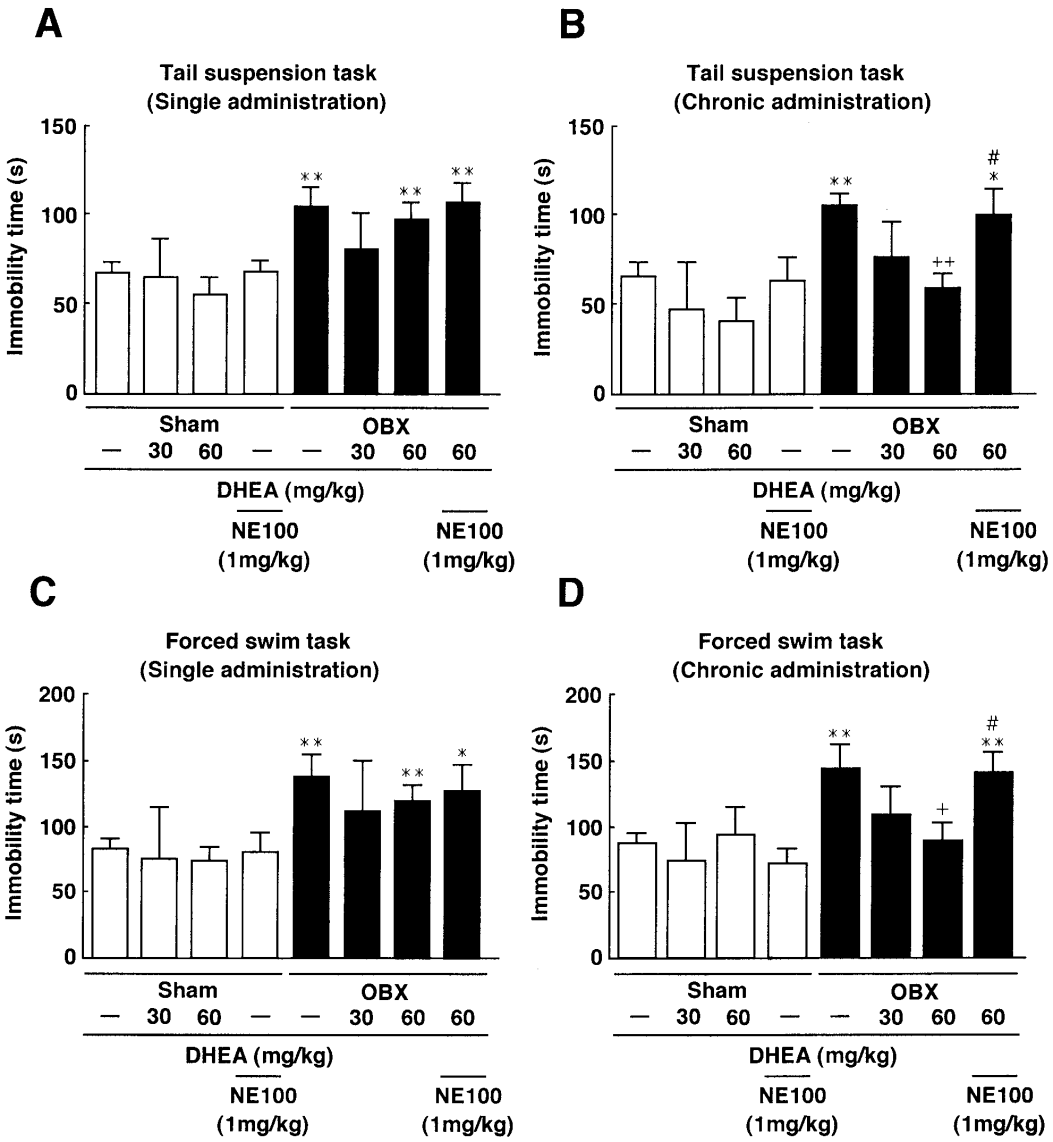
DHEA antagonizes depressive-like behaviors in OBX mice. A: Immobility time in a tail suspension test was measured following a single administration of DHEA at 30 or 60 mg/kg p.o. 14 days after OBX surgery. No difference in immobility time was observed among OBX, or DHEA-treated OBX mice (Mann-Whitney U-test, n = 5 per group). B: Immobility time in the tail suspension test was measured after repeated, chronic administration of DHEA at 30 or 60 mg/kg p.o. for 12–13 days, starting 14 days after OBX surgery. DHEA rescued immobility time in OBX mice. NE-100 (1 mg/kg p.o.) pre-administration significantly inhibited decreased immobility time seen following DHEA treatment of OBX mice (Mann-Whitney U-test, n = 5 per group). C: Immobility time in a forced swim test was measured following a single administration of DHEA at 30 or 60 mg/kg p.o. 14 days after OBX surgery. No difference in immobility time was observed between OBX, or DHEA-treated OBX (Mann-Whitney U-test, n = 5 per group). D: Immobility time in a forced swim test was measured after repeated DHEA administration at 30 or 60 mg/kg p.o. for 12–13 days, starting 14 days after OBX operation. DHEA rescued immobility time seen in OBX mice. Pre-administration of NE-100 (1 mg/kg p.o.) significantly inhibited DHEA-mediated rescue of immobility time in OBX mice (Mann-Whitney U-test, n = 5 per group). Vertical lines show SEM. *, p<0.05; **, p<0.01 versus sham-operated mice. ^+^, p<0.05; ^++^, p<0.01 versus OBX mice. #, p<0.05 versus DHEA-treated OBX mice.

We next asked whether single or repeated treatments with DHEA ameliorated depressive-like behaviors in OBX mice by undertaking tail suspension or forced swim tests. When we measured immobility time following tail suspension, the immobility time significantly increased in OBX compared to sham-operated mice (sham: 66.8±6.3 sec, n = 5; OBX: 104.1±10.6 sec, n = 5), indicative of depressive behavior. Repeated treatment of OBX mice with DHEA at 60 mg/kg significantly decreased immobility time (DHEA: 58.9±7.9 sec, n = 5). Pre-treatment of OBX mice with NE-100 significantly increased immobility time relative to treatment with repeated doses of DHEA alone (NE-100: 99.5±15.3 sec, n = 5) (Fig. 3B). By contrast, a single DHEA treatment did not alter immobility time compared with that seen in OBX mice (Fig. 3A).

Similarly, when we assessed immobility using the forced swim test, OBX mice showed significantly increased immobility times compared to sham-operated mice (sham: 83.5±7.0 sec, n = 5; OBX: 143.9±17.8 sec, n = 5). Repeated DHEA treatments at 60 mg/kg significantly decreased immobility time compared to untreated OBX mice (DHEA: 89.0±14.5 sec, n = 5). Pre-treatment of OBX mice with NE-100 (1 mg/kg) significantly increased immobility time relative to mice that had undergone repeated DHEA treatment (NE-100: 140.3±15.5 sec, n = 5) (Fig. 3D). Finally, similar to results seen in the tail suspension test, in OBX mice a single treatment with DHEA did not alter immobility time in the forced swim test relative to that seen in untreated mice (Fig. 3C).

### DHEA Ameliorates Decreased Hippocampal Neurogenesis Seen in OBX Mice

**Figure 4:**
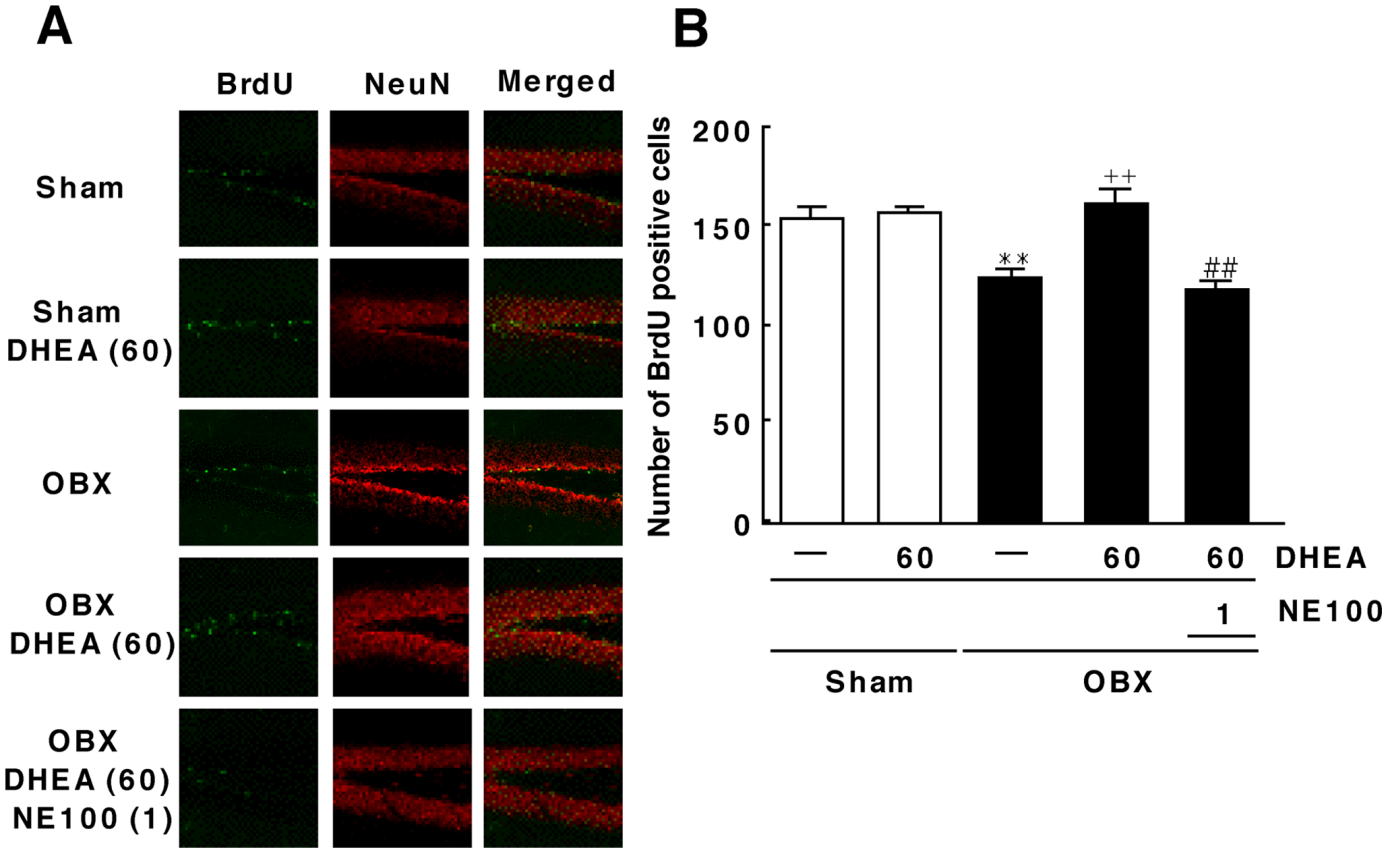
DHEA treatment enhances hippocampal neurogenesis in OBX mice. A: Confocal microscopy images showing double staining for BrdU (green), NeuN (red) and merged images in hippocampal slices 30 days after OBX surgery (n = 8). B: Quantitative analyses of the number of BrdU/NeuN double-positive cells in the DG (n = 8). Vertical lines show SEM. **, p<0.01 versus sham-operated mice. ^++^, p<0.01 versus OBX mice. ##, p<0.01 versus DHEA-treated OBX mice.

Mice were injected with BrdU 20–24 days after OBX or sham surgery and analyzed for hippocampal neurogenesis 7 days later. To identify BrdU-positive cells, hippocampal slices were double-stained with antibodies to BrdU and NeuN, a neuronal marker. Sham-operated mice exhibited a moderate number of BrdU/NeuN double-positive cells in the DG region of the hippocampus (Fig. 4A), but that number significantly decreased in OBX mice (sham: 153.3±5.3 cells, n = 8; OBX: 123.4±3.5 cells, n = 8) (Fig. 4A and 4B). Repeated treatment with DHEA at 60 mg/kg significantly increased the number of BrdU/NeuN double-positive cells in OBX mice relative to untreated controls (160.5±6.9 cells, n = 8) (Fig. 4A and 4B). Pre-treatment of DHEA-treated OBX mice with NE-100 (1 mg/kg) significantly decreased the number of BrdU/NeuN double-positive cells compared with OBX mice receiving DHEA only (117.0±4.3 cells, n = 8) (Fig. 4A and 4B).

### DHEA Treatment Increases Phosphorylation of Akt (Ser-473), Akt (Thr-308) and ERK in the DG of OBX Mice

**Figure 5:**
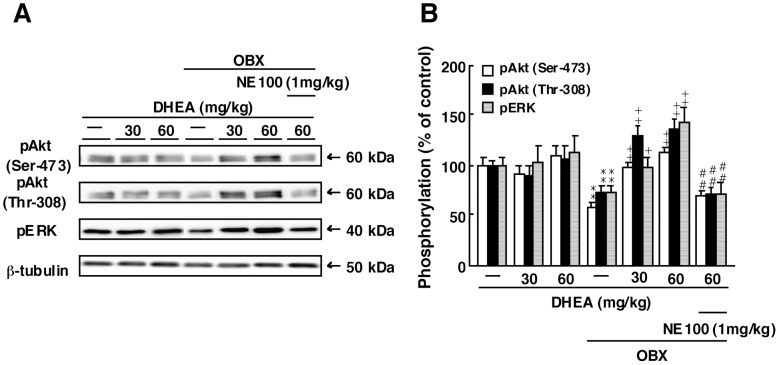
DHEA treatment restores reduced phosphorylation of Akt (Ser-473), Akt (Thr-308) and ERK in the DG of OBX mice. A: Representative images of immunoblots using antibodies against phosphorylated Akt (Ser-473), phosphorylated Akt (Thr-308) and phosphorylated ERK. B: Quantitative analyses of phosphorylated Akt (Ser-473), phosphorylated Akt (Thr-308) and phosphorylated ERK. Vertical lines show SEM. **, p<0.01 versus sham-operated mice. ^+^, p<0.05; ^++^, p<0.01versus OBX mice. ##, p<0.01 versus DHEA-treated OBX mice.

Activation of extracellular signal regulated kinase (ERK) and phosphatidylinositol 3-kinase (PI3K)/Akt pathways in neural precursors plays an essential role in their proliferation and maturation [43]. To determine whether ERK and Akt activities function in DHEA-induced neurogenesis we undertook immunoblot analysis. In sham-operated mice, repeated DHEA administration (30 or 60 mg/kg) had no effect on phosphorylation of Akt (Ser-473), Akt (Thr-308) or ERK in the DG. In OBX mice, phosphorylation of Akt (Ser-473), Akt (Thr-308) and ERK in the DG markedly decreased compared to sham animals (Akt (Ser-473): 58.1±4.6% of sham-operated mice, n = 4; Akt (Thr-308): 74.4±5.1% of sham-operated mice, n = 4; ERK: 73.9±5.6% of sham-operated mice, n = 4). DHEA treatment at 30 or 60 mg/kg significantly increased phosphorylation of Akt (Ser-473), Akt (Thr-308) and ERK in the DG from OBX mice (Akt (Ser-473) (60 mg/kg): 98.4±4.6% of sham-operated mice, n = 4; Akt (Thr-308) (60 mg/kg): 129.2±10.3% of sham-operated mice, n = 4; ERK (60 mg/kg): 143.3±15.2% of sham-operated mice, n = 4) (Fig. 5A and 5B). Pre-treatment of DHEA-treated OBX mice with NE-100 (1 mg/kg) significantly inhibited increased phosphorylation of Akt (Ser-473), Akt (Thr-308) and ERK in the DG relative to mice administered DHEA only (Akt (Ser-473): 69.3±6.2% of sham-operated mice, n = 4; Akt (Thr-308): 70.8±7.6% of sham-operated mice, n = 4; ERK: 70.3±11.9% of sham-operated mice, n = 4) (Fig. 5A and 5B).

### DHEA Treatment of OBX Mice Antagonizes GSK-3β (Ser-9) Phosphorylation and Enhances Neurogenesis

**Figure 6:**
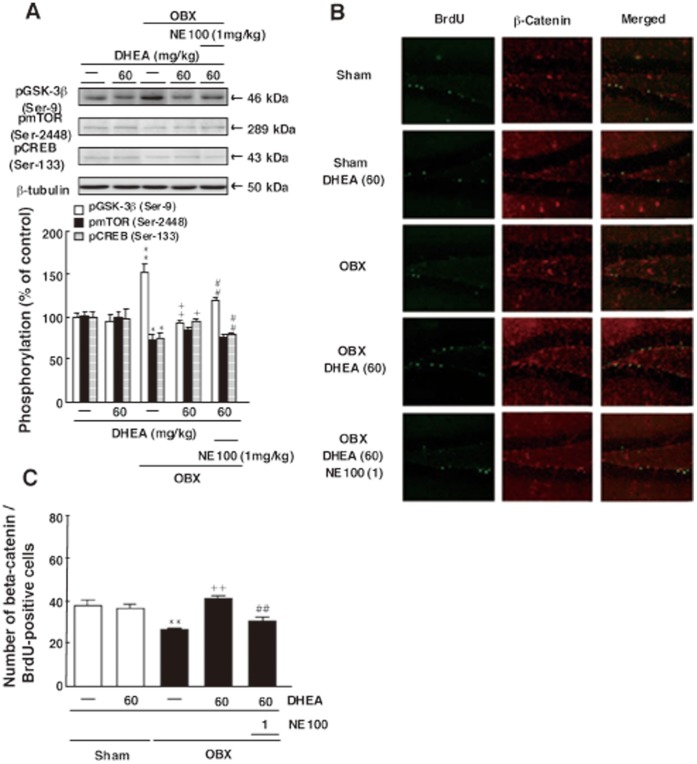
DHEA treatment antagonizes increased GSK-3β (Ser-9) phosphorylation seen in the DG of OBX mice and increases the number of BrdU-positive neurons showing nuclear β-catenin. A: Representative images of immunoblots using antibodies against phosphorylated GSK-3β (Ser-9), phosphorylated mTOR (Ser-2448), phosphorylated CREB (Ser-133) and quantitative analyses of phosphorylated GSK-3β (Ser-9), phosphorylated mTOR (Ser-2448), and phosphorylated CREB (Ser-133). B: Confocal microscopy images showing double staining for BrdU (green), β-catenin (red) and merged images taken from hippocampal slices 30 days after OBX surgery. C: Quantitative analyses of the number of BrdU/β-catenin double-positive cells in the DG. Vertical lines show SEM. **, p<0.01 versus sham-operated mice. ^++^, p<0.01 versus OBX mice. ##, p<0.01 versus DHEA-treated OBX mice.

Finally, stimulation of PI3K/Akt pathways inactivates GSK-3βphosphorylation, promotes nuclear accumulation of β-catenin [44] and also phosphorylates mTOR [45]. Thus we employed immunohistochemical and biochemical analyses to ask whether these activities downstream of PI3K/Akt were required for enhanced hippocampal neurogenesis promoted by DHEA in OBX mice. Sham-operated mice treated with repeated DHEA administration (60 mg/kg) showed no effect on GSK-3β phosphorylation (Ser-9) in the DG compared to untreated mice (Fig. 6A). In OBX mice, GSK-3βphosphorylation (Ser-9) in DG cells markedly increased compared to sham animals (GSK-3β (Ser-9): 152.9±10.1% of sham-operated mice, n = 4). Repeated DHEA treatment at 60 mg/kg significantly decreased GSK-3β phosphorylation (Ser-9) in the DG of OBX mice (GSK-3β (Ser-9): 92.9±3.8% of sham-operated mice, n = 4). Pre-treatment of DHEA-treated OBX mice with NE-100 significantly increased phosphorylation of GSK-3β (Ser-9) in the DG relative to that seen in mice treated with DHEA only (GSK-3β (Ser-9): 119.1±3.6% of sham-operated mice, n = 4) (Fig. 6A). By contrast, OBX mice showed significantly decreased phosphorylation of mTOR (Ser-2448) in DG cells relative to sham animals (mTOR: 73.0±6.1% of sham-operated mice, n = 4). However, DHEA treatment at 60 mg/kg did not alter mTOR phosphorylation in the DG of OBX mice relative to untreated mice (Fig. 6A).

ERK stimulation elevates CREB (Ser-133) phosphorylation in the course of hippocampal neurogenesis [46]. Thus, we asked whether CREB was required for hippocampal neurogenesis in the DG seen following DHEA treatment of OBX mice. In sham-operated mice, repeated DHEA administration (60 mg/kg) had no effects on CREB phosphorylation (Ser-133) in the DG compared to untreated mice. By contrast, OBX mice showed marked decreases in CREB phosphorylation (Ser-133) in the DG relative to sham animals (CREB (Ser-133): 75.7±6.5% of sham-operated mice, n = 4). DHEA treatment at 60 mg/kg significantly restored CREB phosphorylation (Ser-133) in the DG of OBX mice (CREB (Ser-133): 95.1±2.8% of sham-operated mice, n = 4). Pre-treatment of DHEA-treated OBX mice with NE-100 significantly antagonized decreased phosphorylation of CREB (Ser-133) in the DG (CREB (Ser-133): 79.1±3.1% of sham-operated mice, n = 4) (Fig. 6A).

In addition, we also observed BrdU/β-catenin double-positive cells in DG cells from sham-operated mice (Fig. 6B), and that number was significantly decreased in OBX mice (sham: 37.6±2.9 cells, n = 8; OBX: 26.0±10.3 cells, n = 8) (Fig. 6B and 6C). Repeated DHEA treatment at 60 mg/kg significantly increased the number of BrdU/β-catenin double-positive cells in OBX mice relative to untreated OBX mice (40.6±1.1 cells, n = 8) (Fig. 6B and 6C). Pre-treatment of DHEA-treated OBX mice with NE-100 (1 mg/kg) significantly decreased the number of BrdU/β-catenin double-positive cells compared with OBX mice treated with DHEA only (30.8±1.6 cells, n = 8) (Fig. 6B and 6C).

## Discussion

Two different genes encoding sigma receptors have been cloned: sigma-1R and sigma-2R [47]. Sigma-1R and sigma-2R exhibit different molecular weights, 29 kDa and 18–21.5 kDa, respectively [48], [49], [50]. Sigma-1R is widely expressed in both neurons and oligodendrocytes in the CNS and is enriched in the prefrontal cortex, hippocampus and striatum [51], [52], where it functions to regulate inositol 1,4,5-triphosphate receptors and Ca^2+^ signaling in the endoplasmic reticulum (ER) [53]. Stimulation of sigma-1R by various agonists induces translocation of the receptor from the ER membrane to the plasma membrane, thereby regulating ion channel activity and neurotransmitter release [54], [55].

DHEA reportedly interacts with sigma-1R in vivo [56]. We previously reported that stimulation of sigma-1R by DHEA improves cognitive deficits seen in OBX mice. In particular, we observed that in OBX mice DHEA ameliorates LTP deficits in the hippocampal CA1 region by activating CaMKII and PKC [23]. Sigma-1R knockout mice reportedly display depressive-like behaviors when subjected to a forced swim test [57]. Here, we demonstrated that stimulation of sigma-1R by chronic treatment with DHEA improved depressive-like behaviors in OBX mice concomitant with increased neurogenesis in the DG region of the hippocampus.

OBX mice, which serve as an animal model of depressive-like behaviors [7], exhibit down-regulation of the cholinergic system marked by decreased choline acetyltransferase levels in cortex, hippocampus and amygdala [58] and decreased acetylcholinesterase levels in the hippocampus [10]. Interestingly, stimulation of cholinergic system by nicotine ameliorates depressive-like behaviors [59]. Therefore, cholinergic neuronal activity is implicated in adult neurogenesis, and innervation of newborn neurons by cholinergic fibers is seen in the DG [60], [61]. In addition, mechanical lesion of cholinergic neurons suppresses hippocampal neurogenesis in rats [62]. Similarly, agonist activation of sigma-1R induces increases in extracellular acetylcholine (ACh) levels, as measured by microdialysis in vivo [63], and [^3^H] ACh release from the hippocampus [64]. Thus, activation of the cholinergic system by stimulation of sigma-1R by DHEA likely improves depressive-like behaviors through hippocampal neurogenesis.

We also documented that DHEA activation of the sigma-1R stimulates Akt and ERK, and that phosphorylation, and hence activation, of both is decreased in the DG of OBX mice. Activation of these pathways in neural progenitors plays an essential role in their proliferation and maturation [43]. Stimulation of sigma-1R increases calcium transport from the endoplasmic reticulum to mitochondria [65] and enhances N-methyl-D-aspartate receptor (NMDAR)-evoked intracellular Ca^2+^ mobilization in cultured hippocampal neurons [66]. NMDARs are Ca^2+^ permeable ligand-gated ion channels, and Ca^2+^ influx through these ligand-gated receptors is responsible for changes in synaptic plasticity such as LTP [67]. Sigma-1R stimulation also blocks small-conductance calcium-activated potassium channel in the hippocampus [68]. Taken together, membrane depolarization likely triggers PI3K/Akt activation [69]. In fact, here we confirmed that DHEA improves NMDAR-dependent LTP, which is impaired in the DG of OBX mice, through CaMKII activation. In addition, DHEA stimulation of sigma-1R may enhance hippocampal neurogenesis via PI3K/Akt pathways and potentiate synaptic efficacy through CaMKII activation. However, in this study we did not examine the role of endogenous DHEA in regulating sigma-1R affinity in the DG of OBX mice. In fact, sigma-1R expression levels decreased in the DG of OBX mice (data not shown). DHEA is synthesized from cholesterol by *de novo* via 17, 20-desmolase. Future studies will be required to address the role of endogenous DHEA in the DG for hippocampal neurogenesis targeting the inhibition or knockout of 17, 20-desmolase.

Stimulation of PI3K/Akt pathways promotes inactivation of GSK-3β phosphorylation, nuclear accumulation of β-catenin, and mTOR activation [44]. We also found that DHEA treatment antagonized phosphorylation of GSK-3β (Ser−9) seen in untreated OBX mice and that β-catenin co-localized with BrdU in progenitor cells undergoing hippocampal neurogenesis. We did not observe evidence suggesting that mTOR acts a downstream target of PI3K/Akt pathway in enhanced neurogenesis mediated by DHEA. Thus activation of only part of the Akt/GSK-3β/β-catenin pathway likely functions in neurogenesis in the DG. Here, we also demonstrated that activation of the ERK/CREB pathway is also important for hippocampal neurogenesis. Interestingly, reactive oxygen species (ROS) induce hippocampal synaptic efficacy by activating the ryanodine receptor type3 and ERK [70], and activation of PI3K/Akt pathways by membrane depolarization is implicated in ROS generation [69].

In conclusion, this study demonstrates that sigma-1R stimulation by DHEA ameliorates depressive-like behaviors in OBX mice concomitant with increased hippocampal neurogenesis in the DG by activating Akt/GSK-3β/β-catenin pathways. DHEA also improved hippocampal synaptic efficacy in the DG by activating CaMKII. We previously reported that DHEA improves cognitive deficits in OBX mice. Thus, stimulation of sigma-1R may serve as a therapeutic target for both dementia and depression.

## Materials and Methods

### Animals

Adult male DDY mice 8–9 weeks-of-age (Nippon SLC, Hamamatsu, Japan) were housed in cages with free access to food and water at a constant temperature (23±1°C) and humidity (55±5%) with a 12-h light/dark cycle (09:00–21:00 h). All experimental animal procedures were approved by the Committee on Animal Experiments at Tohoku University.

### Operation

OBX mice were prepared as described previously [10]. Mice were treated once a day for 14 days with DHEA starting 14 days after OBX surgery. Behavioral tests were performed 12–13 days after the start of DHEA or DHEA plus NE100 treatment and electrophysiological and biochemical experiments were performed 13–14 days after the start of DHEA treatment. OBX-operated mice showed no stereotyped killing behavior at least until 3 weeks after the operation. However, aggressive behavior without killing was observed in several mice. All animals were sacrificed at the end of experiment and the lesions were verified histologically.

### Tail Suspension Test

The tail suspension test is widely used to assess antidepressant-like activity. The test is based on the fact that animals subjected to the short-term, inescapable stress of being suspended by the tail will develop an immobile posture. The total duration of immobility induced by tail suspension was scored according to the method described by Steru et al. (1985) [35]. In brief, acoustically and visually isolated mice were suspended 50 cm above the floor by adhesive tape placed approximately 1 cm from the tip of the tail. Immobility time was recorded during a 10-min period. Mice were considered immobile only when they hung passively and remained completely motionless.

### Forced Swim Test

Mice were subjected to the forced-swim test at 12–13 days after start of DHEA treatment. Mice were placed individually in glass cylinders (height: 20 cm, diameter: 15 cm) filled 12 cm of water at 25°C. The total duration of immobility within the course of a 5-min test was scored as described by Porsolt et al. (1977) [36]. Mice were judged “immobile” when they ceased struggling and remained floating motionless in the water, making only movements necessary to keep their head above water.

### Electrophysiology

Hippocampal slices were prepared as described [37]. Transverse hippocampal slices (400 µm thick) prepared using a vibratome (Microslicer DTK-1000) were incubated for 2-h in continuously oxygenized (95% O_2_, 5% CO_2_) artificial cerebrospinal fluid at room temperature. Then, slices were transferred to an interface recording chamber and perfused at a flow rate of 2 ml/min with ACSF warmed to 34°C. Field excitatory postsynaptic potentials were evoked by a 0.05 Hz test stimulus through a bipolar stimulating electrode placed on the perforant path from the entorhinal cortex and recorded from the inside granule cell layer using a glass electrode filled with 3 M NaCl. High frequency stimulation of 100 Hz with a 1-s duration was applied twice with a 10-s interval and test stimulation was continued for the indicated periods. After recording, slices were transferred to a plastic plate cooled on ice to dissect out the DG, which were frozen in liquid nitrogen and stored at −80°C until biochemical analysis was performed.

### Biochemical Analysis

Analysis was performed as described previously [37], [38] using the following antibodies: anti-phospho CaMKII, (1∶5000, Fukunaga et al., 2002 [39]), anti-CaMKII, (1∶5000, Fukunaga et al., 1995 [40]), anti-phospho-CaMKIV (Thr-196) (1∶2000, Abcam, Cambridge, MA, USA), anti-phospho-synapsin I (Ser-603) (1∶2000, Millipore, Billerica, MA, USA), anti-synapsin 1 (1∶2000, Fukunaga et al., 1995 [40]), anti-phospho-GluR1 (Ser-831) (1∶1000, Millipore), anti-GluR1 (1∶1000, Millipore), anti-phospho-Akt (Ser-473) (1∶1000, Millipore), anti-phospho-Akt (Thr-308) (1∶1000, Millipore), anti-phospho-MAP kinase (Diphospholated ERK 1/2) (1∶2000, Sigma-Aldrich, St. Louis, MO, USA), anti-phospho-GSK-3β(Ser-9) (1∶1000, Invitrogen, Carlsbad CA, USA), anti-phospho-mammalian target of rapamycin (mTOR) (Ser-2448) (1∶1000, Millipore), anti-phospho- cAMP-responsive element binding protein (CREB) (Ser-133) (1∶1000, Millipore), and anti-β-tubulin (1∶5000, Sigma-Aldrich). Bound antibodies were visualized using the enhanced chemiluminescence detection system (Amersham Life Science, Buckinghamshire, UK) and analyzed semiquantitatively using the National Institutes of Health Image program.

### Immunohistochemistry

Immunohistochemistry was performed as reported by Shioda et al. [14]. Mice were anesthetized with sevoflurane and perfused via the ascending aorta with phosphate-buffered saline (PBS; pH 7.4) until the outflow became clear. At that time, the perfusate was switched to phosphate buffer (pH 7.4) containing 4% paraformaldehyde for 15 min. The brain was removed, post-fixed in the same solution for 24-h at 4°C, and sliced at 50 µm using a vibratome (Dosaka EM Co. Ltd., Kyoto, Japan). Coronal brain sections were incubated as follows: 30 min in PBS; 30 min 2 N HCl; 1-h in PBS with 3% bovine serum albumin (blocking solution); overnight with mouse anti-NeuN monoclonal antibody (1∶500) (Millipore), a rat anti-bromodeoxyuridine (BrdU) monoclonal antibody (1∶500) (Accurate Chemical and Scientific, Oxford Biotechnology, Oxfordshire, UK), or a mouse anti-β-catenin monoclonal antibody (1∶1000) (MBL, Woods Hole, MA, USA) in blocking solution at 4°C. After thorough washing in PBS, sections were incubated 3-h in Alexa 488-labeled anti-rat IgG (anti-BrdU) or Alexa 594-labeled anti-mouse IgG (anti-β-catenin or anti-NeuN). After several PBS washes, sections were mounted on slides with Vectashield (Vector Laboratories, Burlingame, CA, USA). Immunofluorescent images were analyzed using a confocal laser scanning microscope (Nikon EZ-C1, Nikon, Tokyo, Japan). To count BrdU and NeuN double-positive cells after immunohistochemistry, six hippocampal sections were cut every 50 µm beginning at 1.7 to 2.2 mm caudal to the bregma. The number of BrdU/NeuN or BrdU/β-catenin double-positive cells was determined in a 300×300 µm area per section in the DG region. In the DG, the GCL (approximately 50 µm wide) and the SGZ, defined as a zone two cell bodies wide (5 µm) along the border of the GCL and hilus, were quantified together. The number of BrdU/NeuN or BrdU/β-catenin double-positive cells counted per mouse was expressed as the number of the double-positive cells per a 300×300 µm area. Six sections per mouse and six mice per condition were used. The person responsible for cell counts was blind to the experimental conditions.

### Other Chemicals

DHEA and BrdU were purchased from Sigma-Aldrich (Tokyo, Japan). NE-100 was from Santa Cruz Biotechnology, Inc. (Santa Cruz, CA, USA).

### Data Analysis

Comparison between two experimental groups was made using the unpaired Student’s *t*-test. Statistical significance for differences among groups was tested by one-way analysis of variance (ANOVA), followed by multiple comparisons between control and other groups using Scheffe’s test by StatView (Hulinks, Inc). *P*<0.05 was considered significant.
