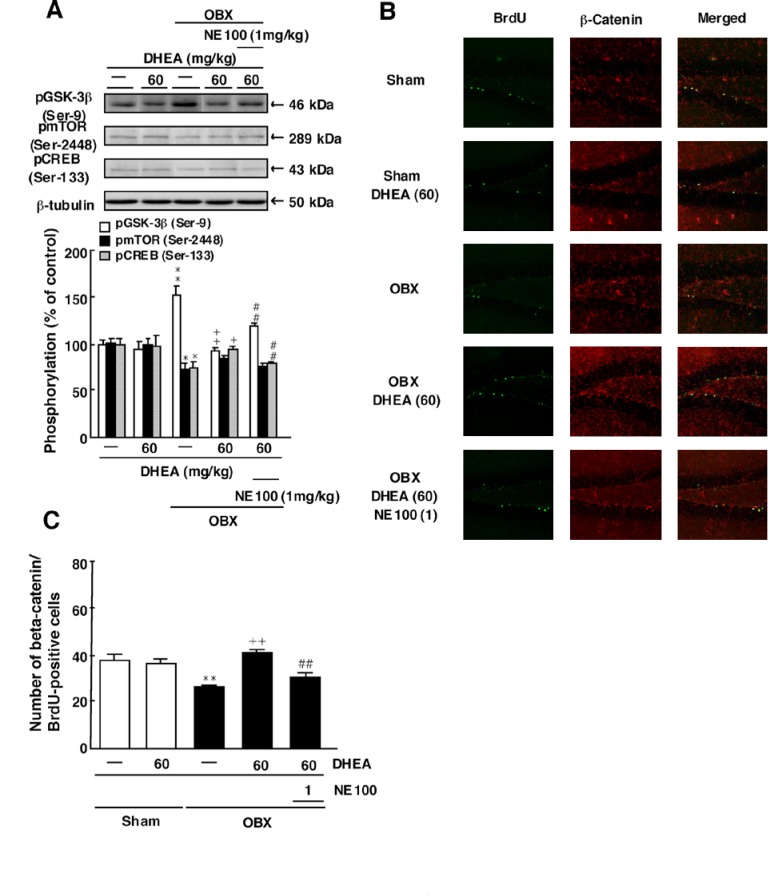# Correction: Stimulation of the Sigma-1 Receptor by DHEA Enhances Synaptic Efficacy and Neurogenesis in the Hippocampal Dentate Gyrus of Olfactory Bulbectomized Mice

**DOI:** 10.1371/annotation/9fdc3705-7112-4382-a3a2-dcde33229272

**Published:** 2014-01-08

**Authors:** Shigeki Moriguchi, Yasuharu Shinoda, Yui Yamamoto, Yuzuru Sasaki, Kosuke Miyajima, Hideaki Tagashira, Kohji Fukunaga

Figure 6 is incorrect. Please view the correct Figure 6 here: 

**Figure:**