# Expression of Concern: Stimulation of the Sigma-1 Receptor by DHEA Enhances Synaptic Efficacy and Neurogenesis in the Hippocampal Dentate Gyrus of Olfactory Bulbectomized Mice

**DOI:** 10.1371/journal.pone.0290363

**Published:** 2023-08-15

**Authors:** The PLOS ONE Editors

After this article [1, 2] was published, concerns were raised about Figures 2, 5, and 6. Specifically, there appear to be multiple instances of vertical discontinuities between lanes in the majority of western blot panels in Figures 2A, 5A, and 6A, and the vertical discontinuities do not appear to be in consistent positions across all panels within each Figure.

In response to queries about the experiments in Figures 2A, 5A, and 6A in [1, 2], the first author provided the original underlying blots for Figure 6A (S1 File), which showed that western blots were performed separately for each protein of interest, that the bands within each panel in Figure 6A are from the same blot, and that multiple lanes have been spliced out in inconsistent positions across all panels. The first author also provided the available individual-level data from which they generated the charts in Figures 2B, 5B, and 6A (S2 File), but stated that the original uncropped and unadjusted image files captured at the time of the experiment for Figures 2A and 5A are no longer available.

The first author also provided some information on the methodological details of the western blot procedures. They stated that western blots were performed separately to detect individual antibodies, with separate samples on separate gels, the protein samples were loaded into each lane of the gel (n = 4 in each group, using 20 (Figs. 2 and 6) or 24 (Fig. 5) lanes), and the bound antibodies were visualized using an enhanced chemiluminescence detection system and analysed semi-quantitatively using ImageJ [5]. They also noted that no blots were stripped and re-probed.

The first author also provided some information on the quantification method for analyzing protein phosphorylation percentage. They stated that they divided the intensity of each protein band by the mean band intensity value of the sham (control) group and the calculated value for each sample was then expressed as the ratio change relative to the sham group. The first author stated that the values for protein phosphorylation were determined by calculating phospho-protein/total protein ratios (Figure 2) or calculating phospho-protein/β-tubulin ratios (Figures 5 and 6) using normalized values, and the resulting ratios for each sample were expressed as percentage changes of the sham group. The quantitative data have not been provided for some of the values used in the normalization calculations (CaMKII, Syn I and GluR1 total proteins and β-tubulin in Figure 2A, and β-tubulin in Figures 5A and 6A).

The Funding Statement for this article [1, 2] states that the study received funding from the Smoking Research Foundation, which according to [3] has received financial support from the tobacco industry. In light of this issue the *PLOS ONE* article [1, 2] does not comply with the journal’s policy on Funding from Tobacco Companies [4] which was implemented in 2010. We regret that this concern was not identified and addressed prior to the article’s [1, 2] publication.

In light of the above concerns with Figures 2A, 5A, and 6A which question the validity of these data, and in the absence of some of the underlying data required to resolve these concerns, the *PLOS ONE* editors issue this Expression of Concern.

## Supporting information

S1 File Underlying western blots for Figure 6A. (PDF)

S2 File Individual-level underlying data for Figures 2B, 5B, and 6A. (PPT)
