# Supplementary figures and images for: Expression of Concern: Stimulation of the Sigma-1 Receptor by DHEA Enhances Synaptic Efficacy and Neurogenesis in the Hippocampal Dentate Gyrus of Olfactory Bulbectomized Mice
Source: PLoS One. 2023 Aug 15;18(8):e0290363. doi: 10.1371/journal.pone.0290363 (PMC10426955; doi:10.1371/journal.pone.0290363)

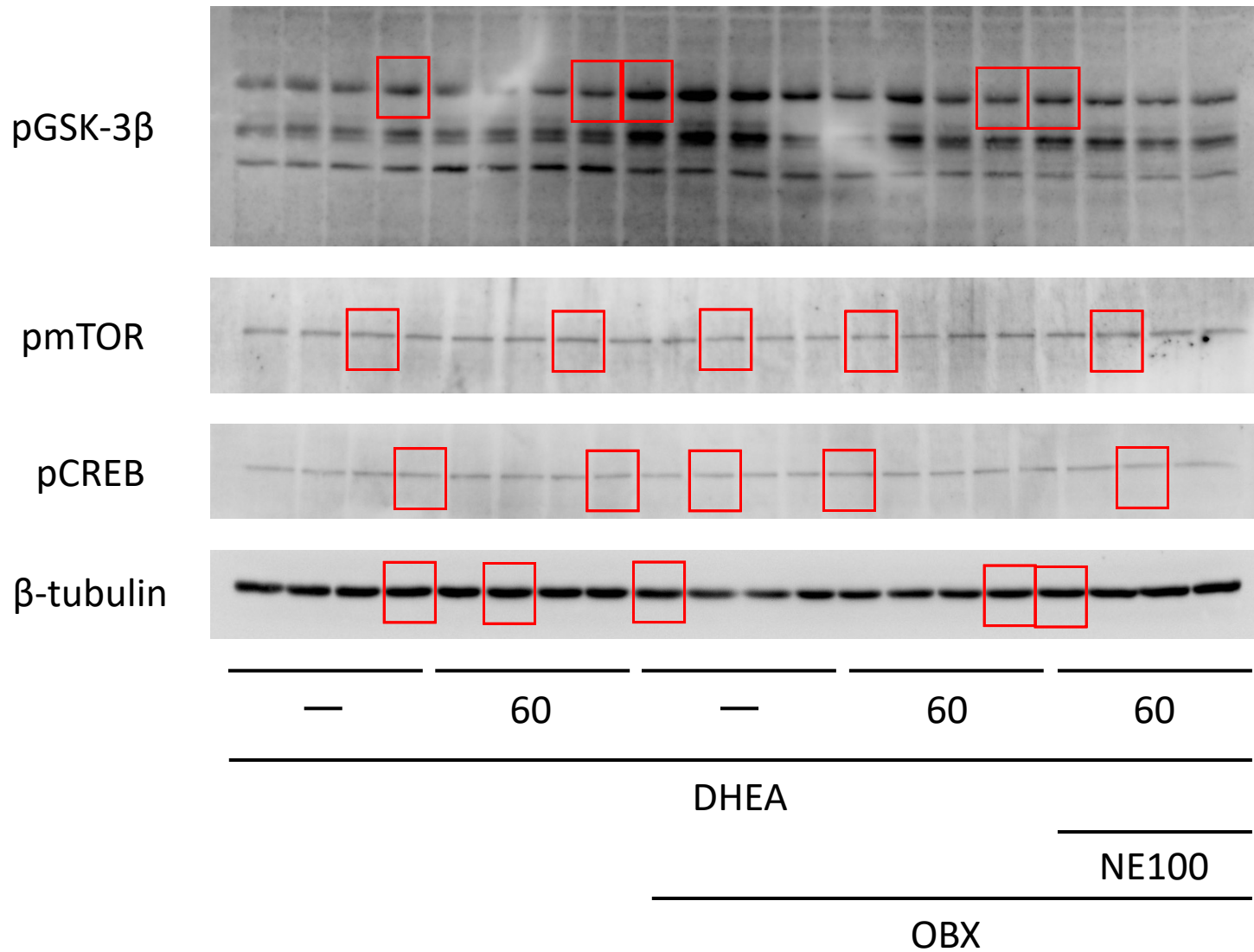

Original data of Figure 6

Supplement: S1 File — (PDF) [file pone.0290363.s001.pdf]
