# Supplementary material for: Expression of Concern: Stimulation of the Sigma-1 Receptor by DHEA Enhances Synaptic Efficacy and Neurogenesis in the Hippocampal Dentate Gyrus of Olfactory Bulbectomized Mice
Source: PLoS One. 2023 Aug 15;18(8):e0290363. doi: 10.1371/journal.pone.0290363 (PMC10426955; doi:10.1371/journal.pone.0290363)
Supplement: S2 File — (PPT) [file pone.0290363.s002.ppt]

## Slide 1
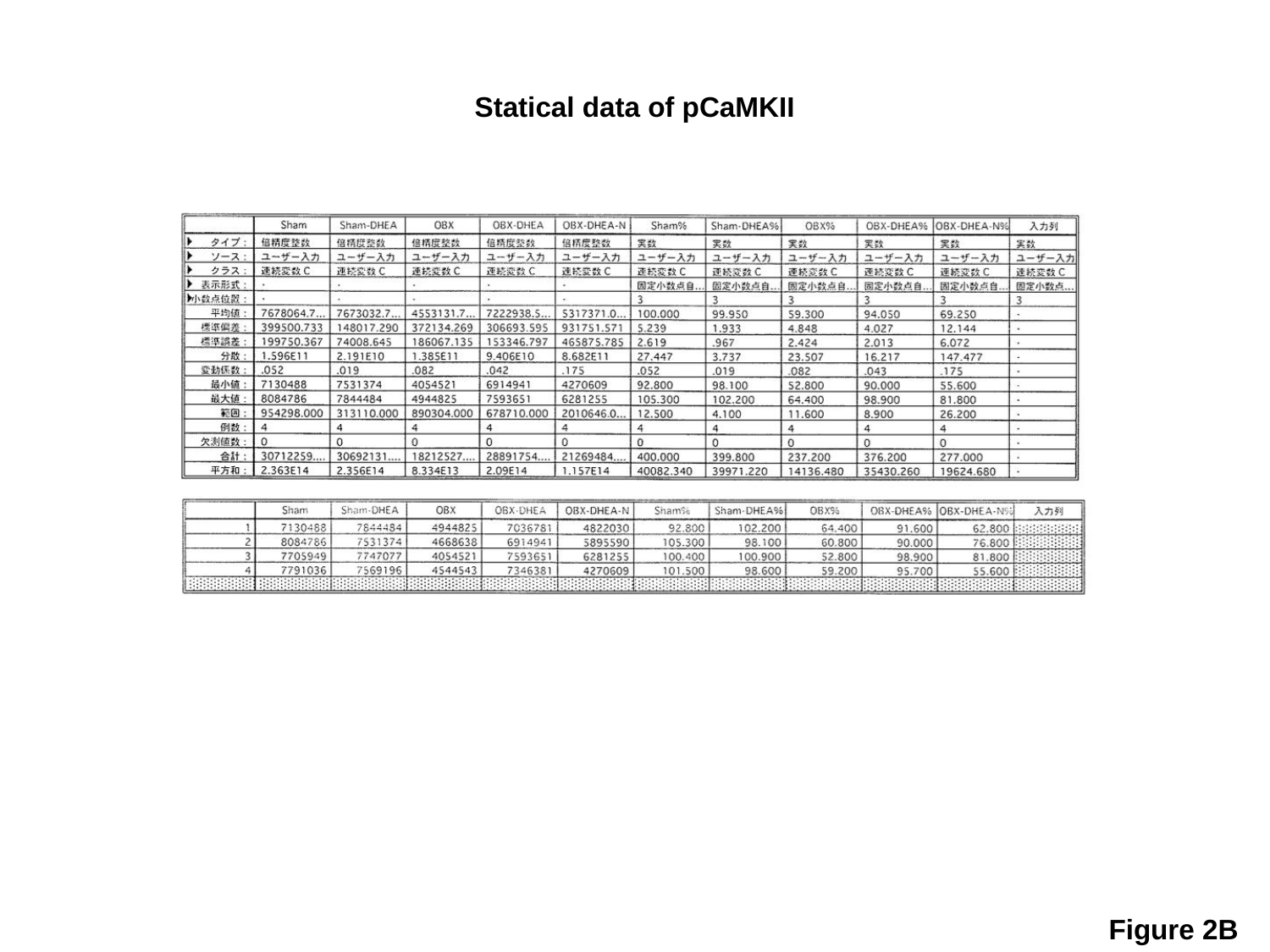

Statical data of pCaMKII
Figure 2B

## Slide 2
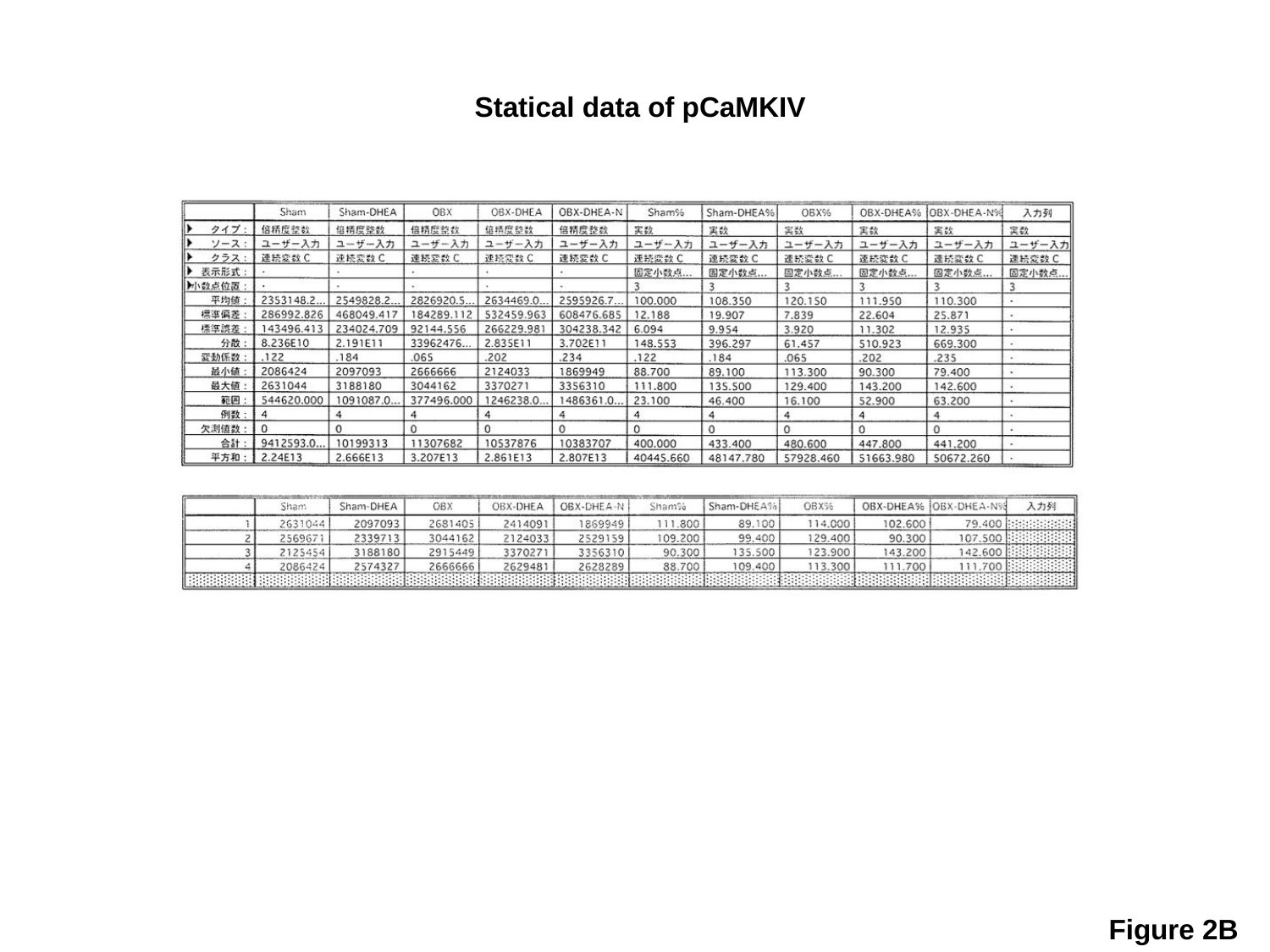

Statical data of pCaMKIV
Figure 2B

## Slide 3
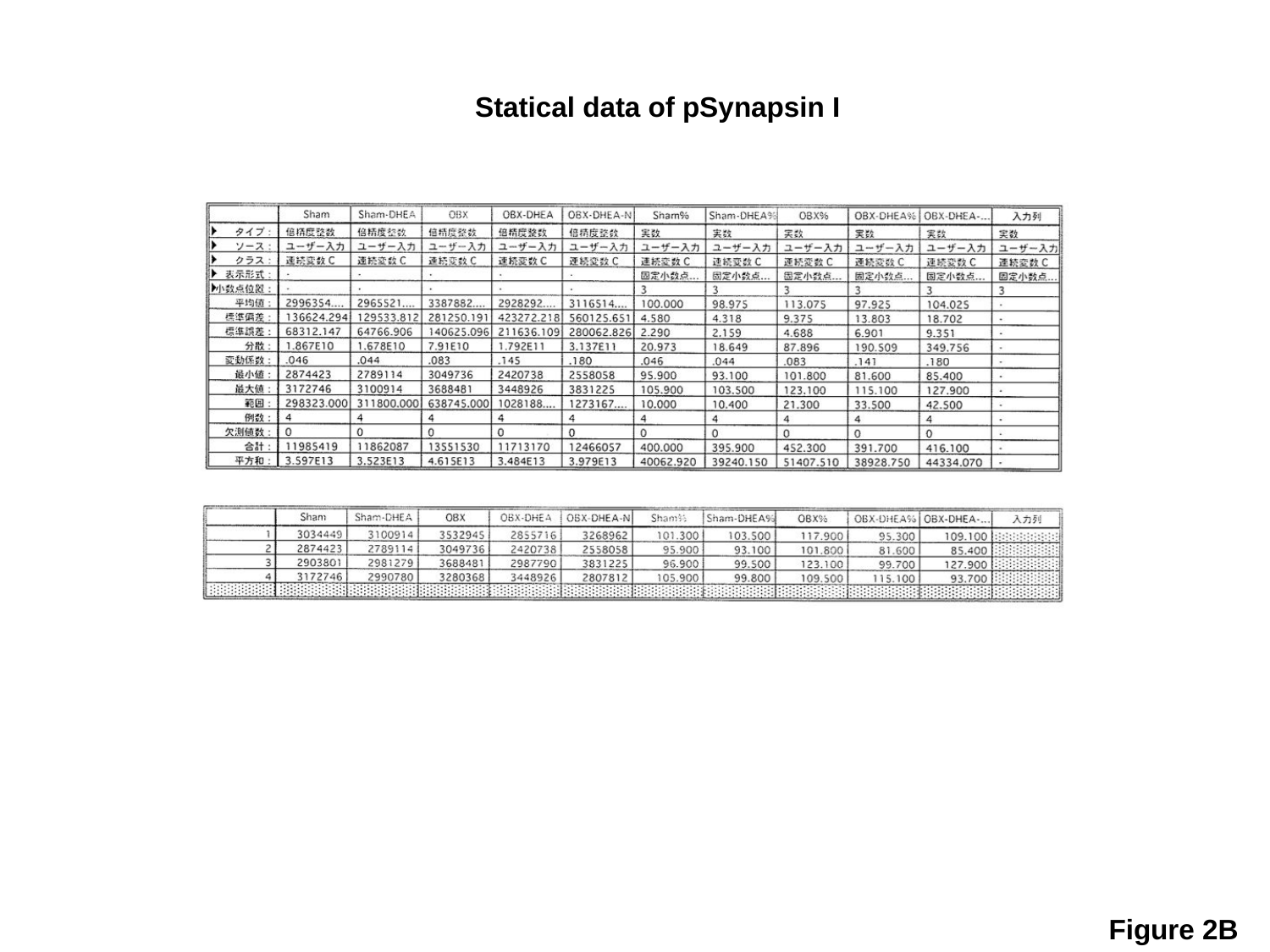

Statical data of pSynapsin I
Figure 2B

## Slide 4
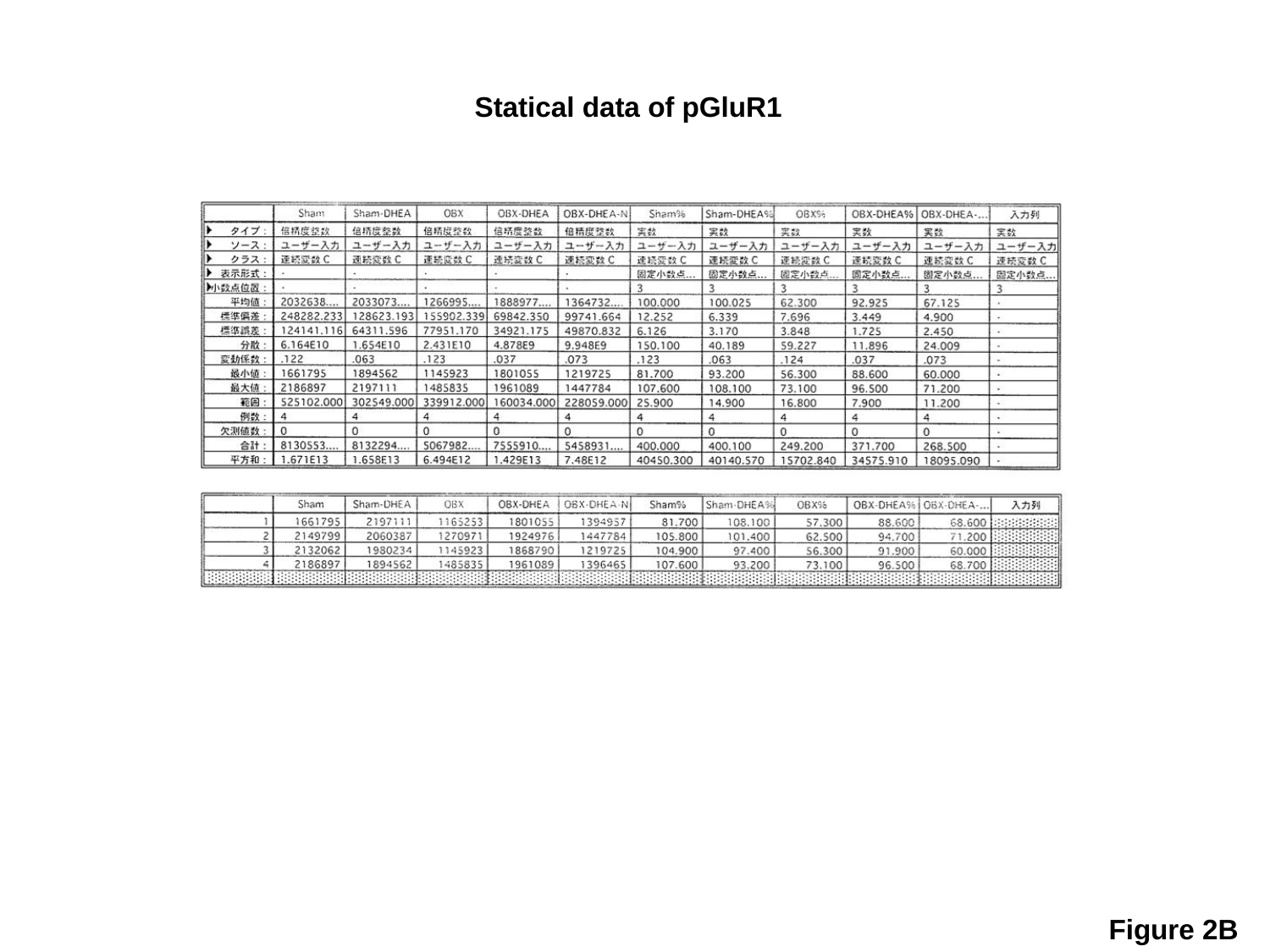

Statical data of pGluR1
Figure 2B

## Slide 5
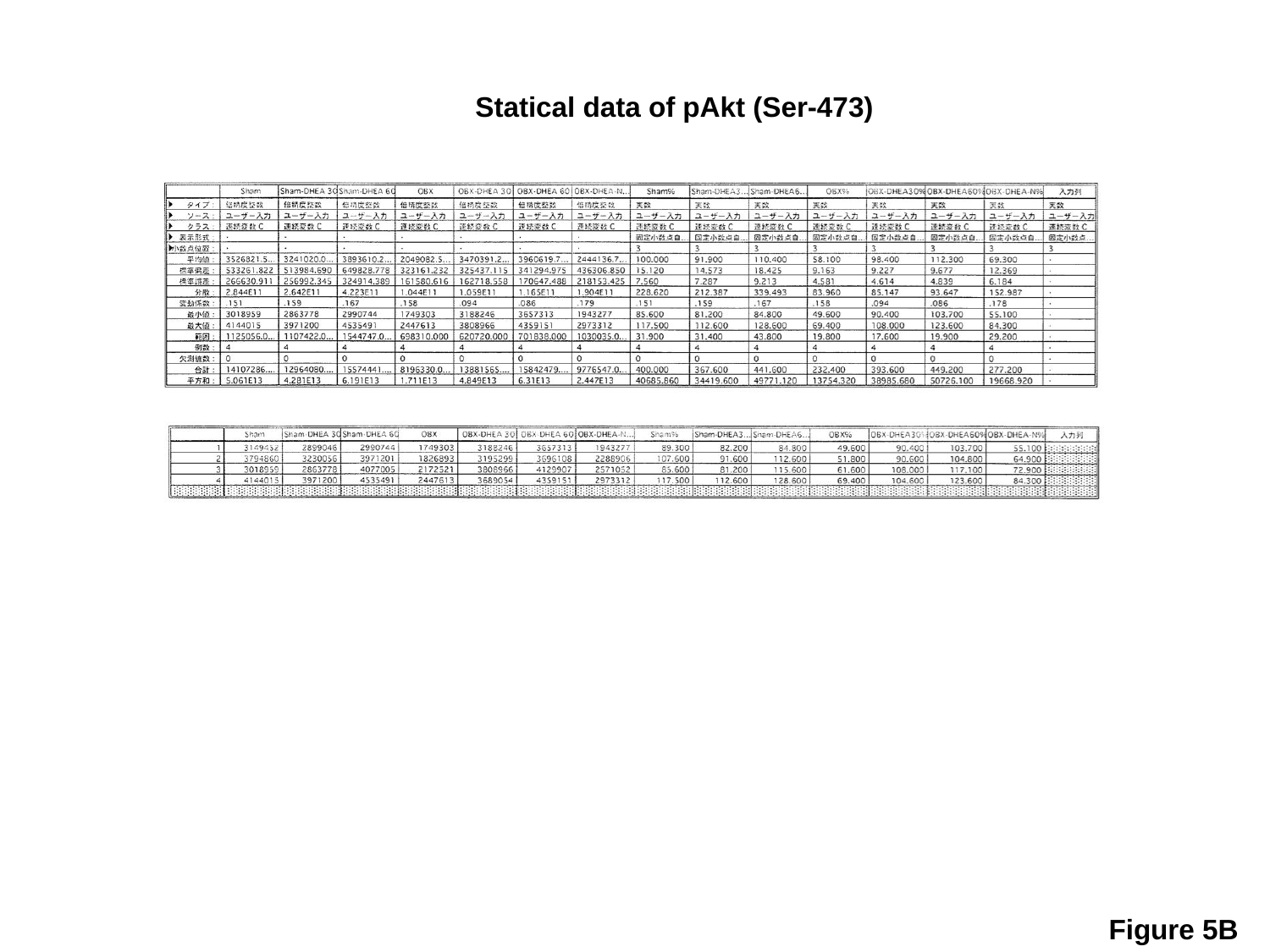

Statical data of pAkt (Ser-473)
Figure 5B

## Slide 6
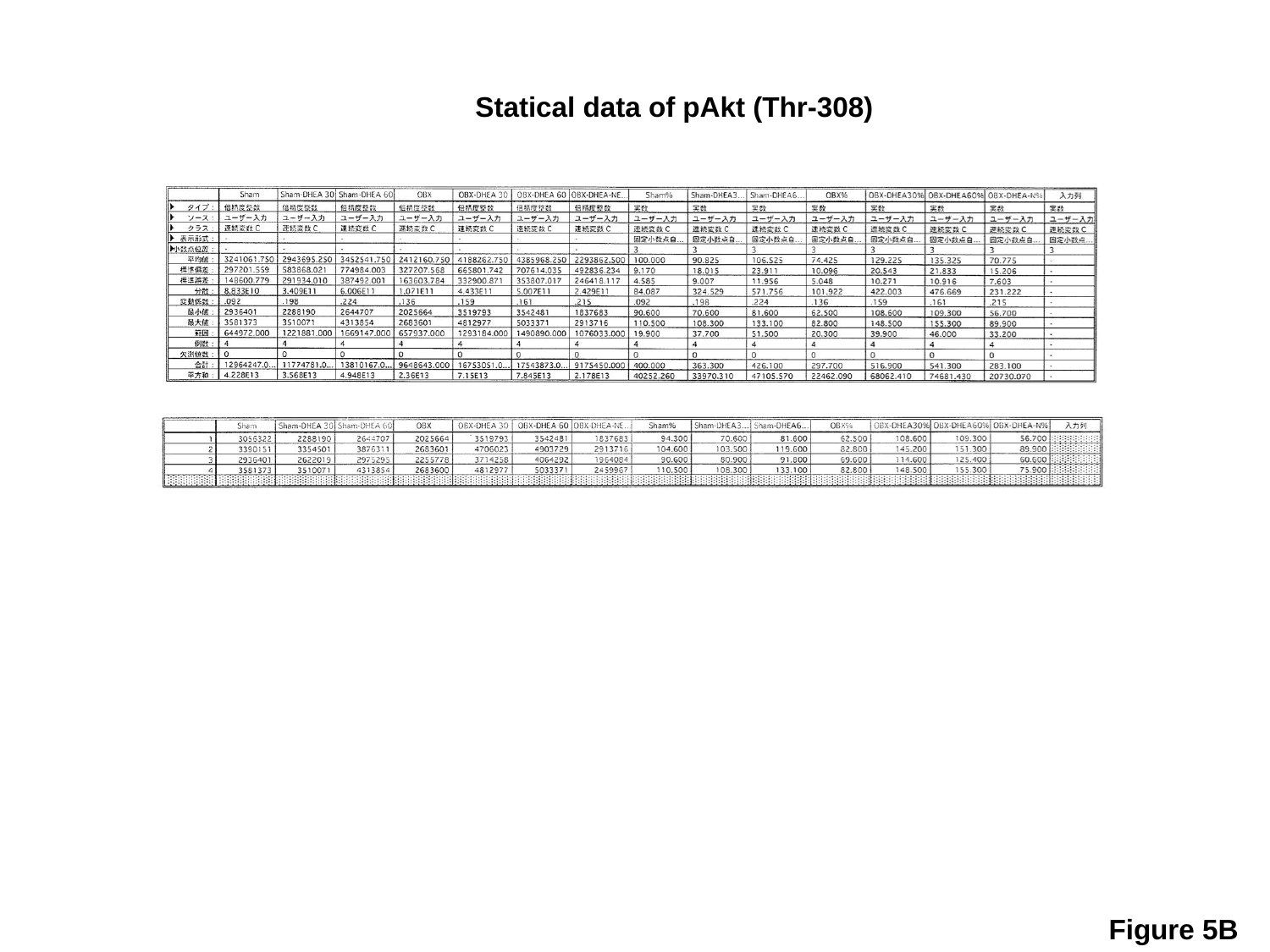

Statical data of pAkt (Thr-308)
Figure 5B

## Slide 7
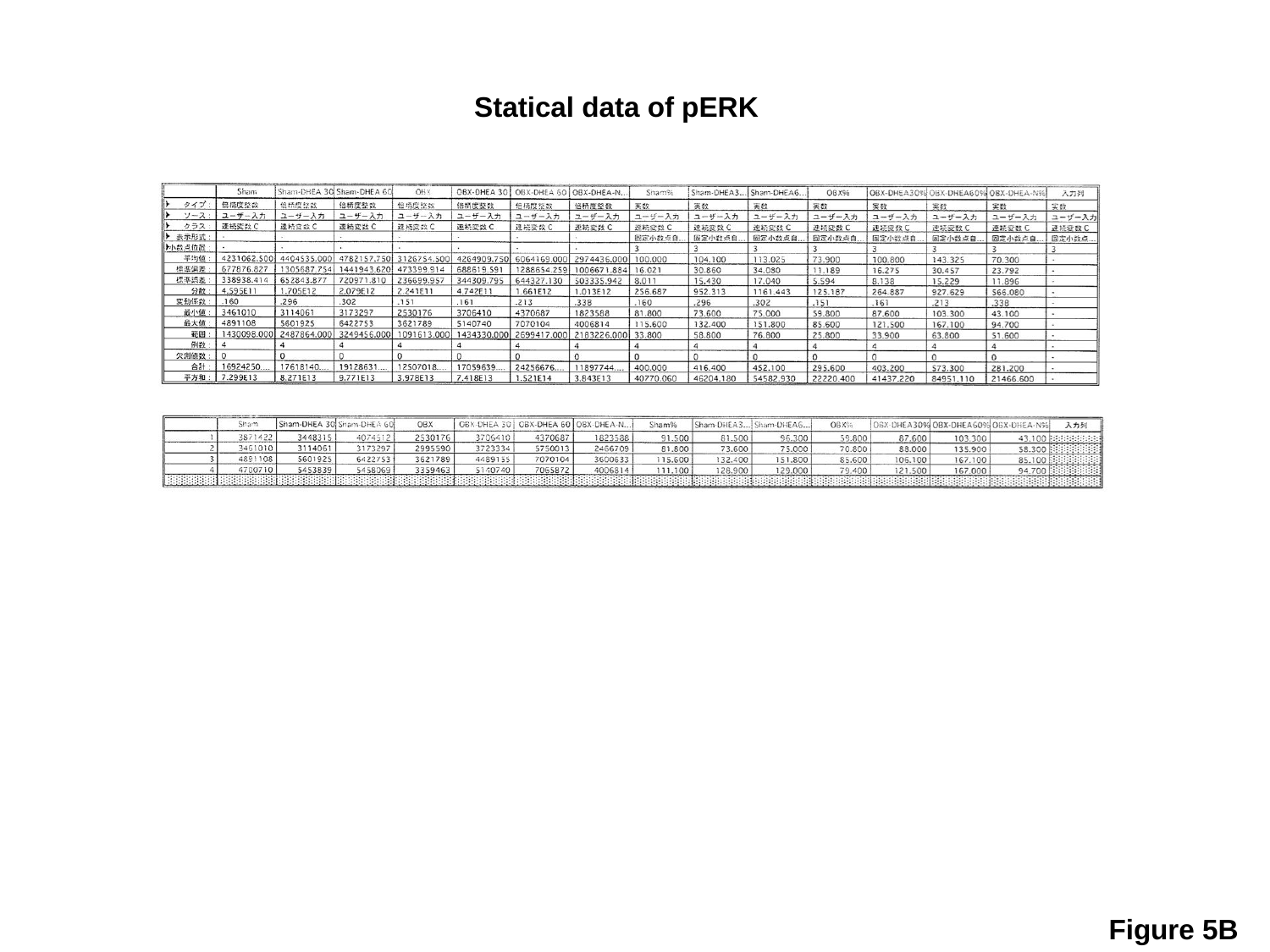

Statical data of pERK
Figure 5B

## Slide 8
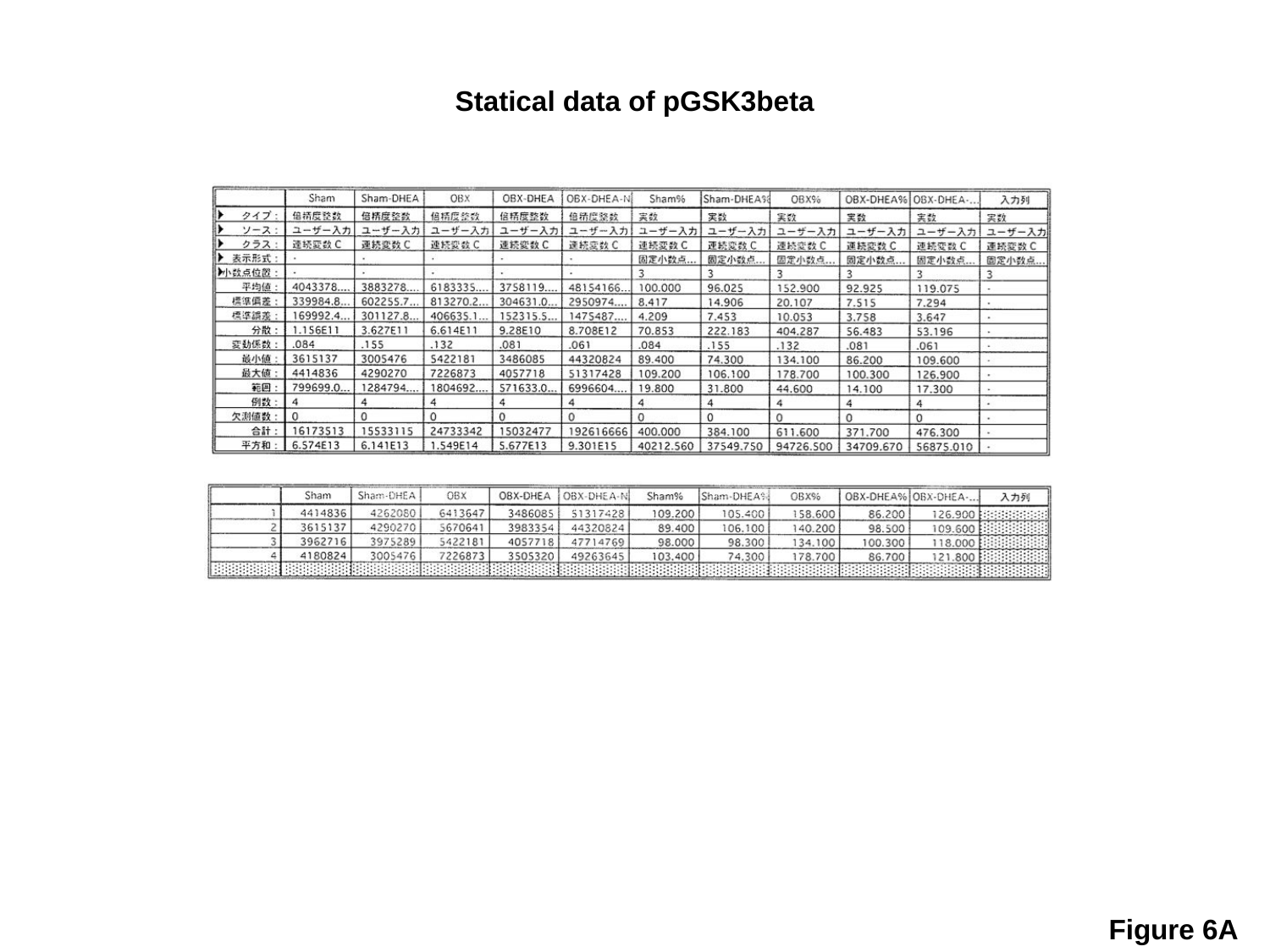

Statical data of pGSK3beta
Figure 6A

## Slide 9
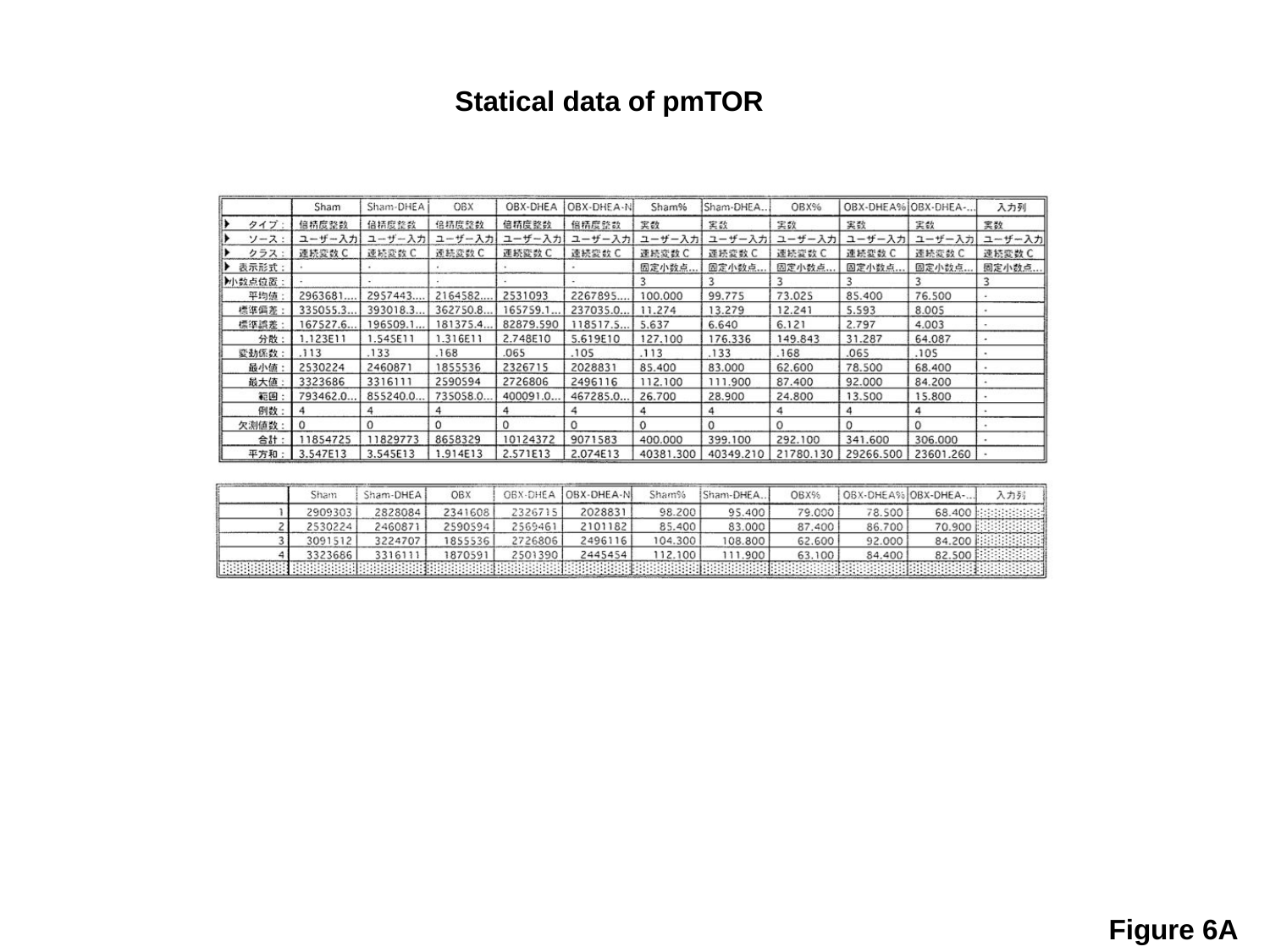

Statical data of pmTOR
Figure 6A

## Slide 10
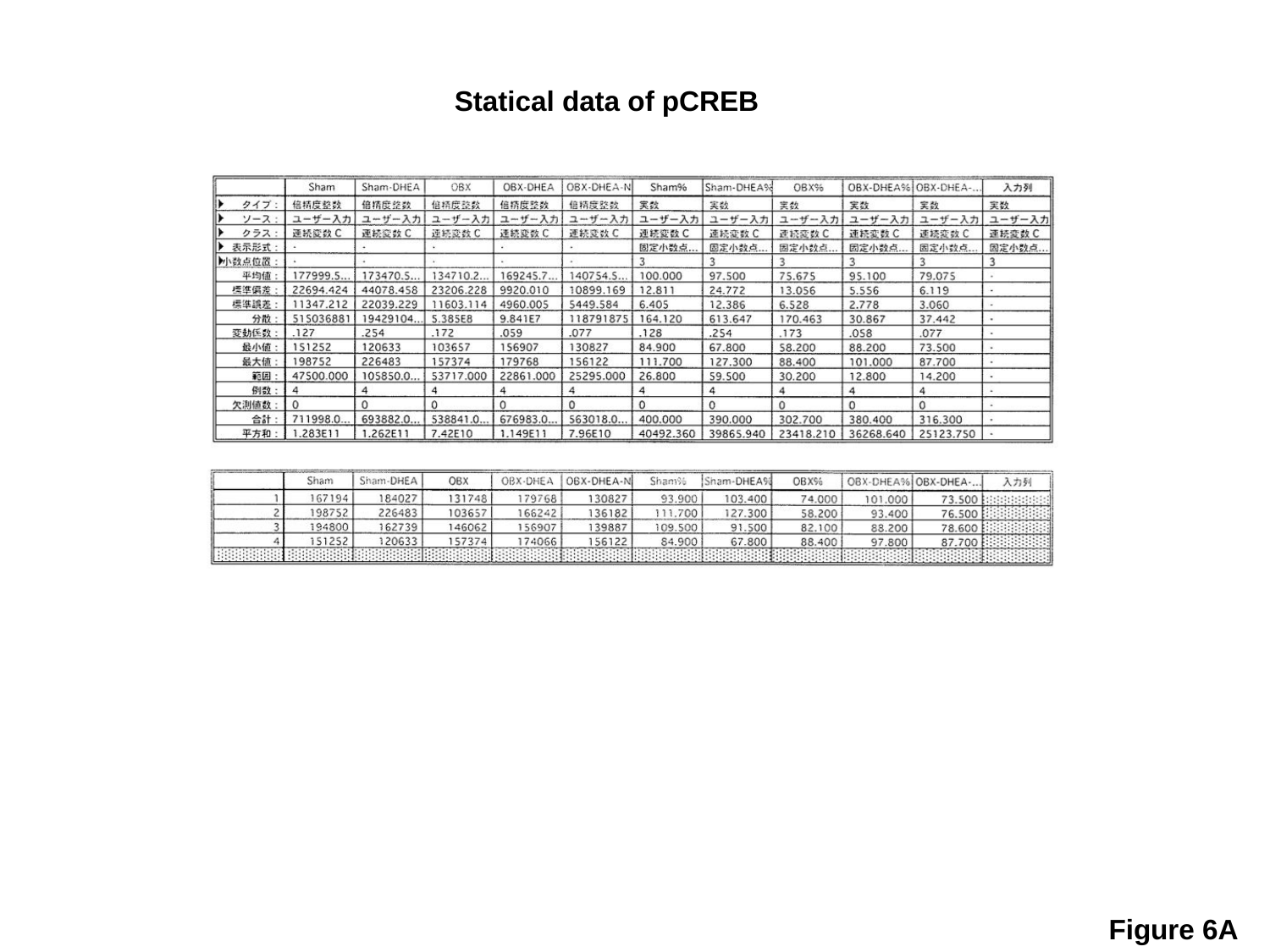

Statical data of pCREB
Figure 6A
